# A Novel Sonification Approach to Support the Diagnosis of Alzheimer’s Dementia

**DOI:** 10.3389/fneur.2017.00647

**Published:** 2017-12-07

**Authors:** Letizia Gionfrida, Agnieszka Roginska

**Affiliations:** ^1^Centre of Performance Science, Royal College of Music, London, United Kingdom; ^2^BICV Group, Bioengineering Department, Faculty of Engineering, Imperial College London, London, United Kingdom; ^3^Music and Audio Research Laboratory, Music Technology, New York University, New York, NY, United States; ^4^Division of Nuclear Medicine, Department of Radiology, NYU School of Medicine, New York, NY, United States

**Keywords:** sonification, medical imaging, PET scan, Alzheimer’s dementia, computer-aided diagnosis

## Abstract

Alzheimer’s disease is the most common neurodegenerative form of dementia that steadily worsens and eventually leads to death. Its set of symptoms include loss of cognitive function and memory decline. Structural and functional imaging methods such as CT, MRI, and PET scans play an essential role in the diagnosis process, being able to identify specific areas of cerebral damages. While the accuracy of these imaging techniques increases over time, the severity assessment of dementia remains challenging and susceptible to cognitive and perceptual errors due to intra-reader variability among physicians. Doctors have not agreed upon standardized measurement of cell loss used to specifically diagnose dementia among individuals. These limitations have led researchers to look for supportive diagnosis tools to enhance the spectrum of diseases characteristics and peculiarities. Here is presented a supportive auditory tool to aid in diagnosing patients with different levels of Alzheimer’s. This tool introduces an audible parameter mapped upon three different brain’s lobes. The motivating force behind this supportive auditory technique arise from the fact that AD is distinguished by a decrease of the metabolic activity (hypometabolism) in the parietal and temporal lobes of the brain. The diagnosis is then performed by comparing metabolic activity of the affected lobes to the metabolic activity of other lobes that are not generally affected by AD (i.e., sensorimotor cortex). Results from the diagnosis process compared with the ground truth show that physicians were able to categorize different levels of AD using the sonification generated in this study with higher accuracy than using a standard diagnosis procedure, based on the visualization alone.

## 1 Introduction

The word dementia indicates a collective group of neurodegenerative disorders characterized by impairment of cognitive and functional abilities together with behavioral symptoms (1). It is also characterized by loss of neurons and synapses, atrophy of brain regions (particularly in the temporal and parietal lobes), and metabolic dysfunction of brain activity. Among different forms of dementia, Alzheimer’s disease is a progressive neurodegenerative disease that accounts for around 80% of dementia cases (2), and it is characterized by difficulties with thinking and, in more severe cases, loss of self-awareness. This is reflected in a decreased metabolic activity (hypometabolism) in the parietal and temporal lobes of the brain (3), while more severe cases also present hypometabolism in the frontal lobe of the brain (4).

**Figure 1:**
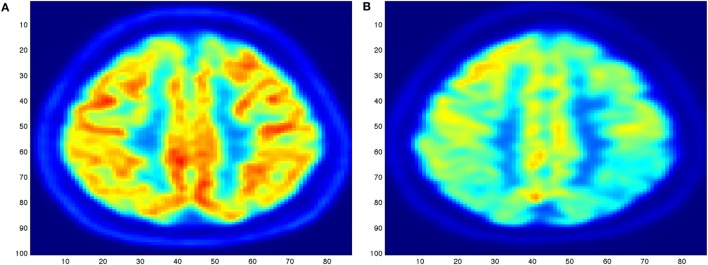
Illustration of an orthogonal PET/CT brain scan obtained with the MIM (19) Inc. to highlight the different levels of activity for a brain not affected **(A)** and for a brain severely affected **(B)** by Alzheimer’s disease.

The diagnosis of AD (2) can definitely be diagnosed after death, with a histopathologic confirmation, but it can be assessed using neuroimaging techniques. Alzheimer’s is also diagnosed based on the person’s medical history, laboratory assessments, and behavioral observations. In particular, medical neuroimaging refers to several different techniques that are used to produce different visualizations of human bodies, tissues, and organs. A standard AD procedure to exclude other cerebral pathology or subtypes of dementia often includes functional and structural imaging techniques as magnetic resonance imaging (MRI) and computed tomography (CT), but such techniques are not able to detect the stages of the disease, especially due to volume loss is not apparent early in the course of the disease (5). In addition to structural imaging, molecular imaging techniques as positron emission tomography (PET) helps in differentiating dementia syndromes for their peculiarity of reflecting cerebral metabolic rates of the brain. Figure 1 shows an orthogonal PET/CT brain scan with a brain not affected and a brain affected by AD. In addition hybrid nuclear/structural imaging analysis, as PET-MRI and PET-CT, have shown the potential to further improve image registration and reduced radiation exposure (6). The advantages of these hybrid techniques are the high resolution and sensitivity, and the simultaneous acquisition of different brain areas (7).

These methods are reliable and advanced during the diagnosis process, but there is still large inconsistency in the diagnosis process among physicians upon different visual inspections provided by these medical imaging (8). The main problem is that the diagnosis is highly dependent on subjective judgment (9) and clinicians continue to have difficulties when the visual analysis leads to imperceptible differences between health and disease (8).

These problems have led physicians and scientists to investigate for assistive diagnosis procedures that together with standard visualizations from imaging techniques can convey information in a supportive way that can aid the diagnosis process (10). Several studies have explored the possibility of aided diagnosis using auditory feedback in medical domains.

The technique that involves non-verbal information that can be transformed into audio feedback and used to facilitate data communication and/or interpretation is called sonification (11). A broad variety of sonification designs have been explored in the past two decades; work such as Walker and Kramer (12) and Walker and Mauney (13), have been successfully employed for esthetic representations, while for Polli (14, 15) have used sonification to describe atmospherically/geographical data. Particularly, expression of medical data into audio domains as supplementary tool has been largely explored, i.e., to inspect multivariate medical data as EEG data (16), or as a supporting method for medical imaging in the diagnosis process. Starting from the assumption that radiologists have to cost their efforts to investigate these medical images (17) used a large amount of MR images providing pieces of evidence that sonification helps in attention rousing and fatigue reduction during medical imaging diagnosis.

In spite of the success in applying auditory information to the previous mentioned datasets, no work has been conducted on the use of sonification to facilitate AD diagnosis process using PET/CT scan. In light of the challenges in diagnosis at assessing Alzheimer’s diagnosis in living patients, we explore a novel sonification tool that enhances the diagnosis of dementia using a PET/CT scan. In this study, we present a sonification tool that improves diagnosis accuracy. We investigate the diagnosis of different stages of AD using a PET/CT scan and how the use of a sonification method added upon the visual inspection can increase accuracy in distinguish between brains of different levels of AD.

## 2 Materials and Methods

The sonification technique to enhance the diagnosis of dementia used a hybrid PET/CT scan for the acquisition process of the medical dataset. Once acquired, the medical dataset was pre-processed by medical physicians to standardize the output to be analyzed. After the pre-processing session, the dataset was analyzed using a tool that allowed the sonification of the medical dataset.

The sonification technique implemented in this tool is called Triple-Tone Sonification (TTS). It aims to enhance explicitness in the diagnosis process taking advantages from the fact that the human ear, as investigated in Ref. (18) article, has a distinct advantage in hearing two close frequencies beating against each other in a phenomenon known as frequency beating. Following the TTS technique, the metabolic activity of the segmented lobes was mapped to the frequency that creates the tone. Particularly, brain lobes detected with a PET/CT scan was segmented, using the MIM (19), into three key areas: frontal lobe, parietal lobes, which are highly affected by AD (4), and sensorimotor cortex (SMC), that usually remains unaffected (2). Each region was then mapped to a different audible frequency and the interaction of these tones’ frequencies resulted in beating patterns. From the acquisition to the sonification, the pre-processing to the analysis, the entire methodology of the PET/CT scan for the 32 de-identified human brains is explained in the following subsections.

### 2.1 Dataset and PET/CT Pre-processing

The dataset used to evaluate the performances of the TTS was obtained from the radiology department of the New York University Langone Medical Center. It consisted of 32 de-identified PET/CT scans of human brains that were diagnosed with different stages of Alzheimer’s dementia. The entire dataset was acquired using the vendor-neural (19) medical software that allows the visualizations of general nuclear medicine imaging. The collection of 32 brain scans was distributed as of eight brain scans in each of four different categories of AD. These categories included eight subjects’ brain not influenced by dementia, eight subjects mildly affected by Alzheimer’s, eight brains with moderate level of AD, and eight subjects’ brain severely affected by Alzheimer’s dementia.

Given the nature of the image-based diagnosis process, as common particle in biomedical investigation, the gold standard (such as the presence of AD and the eventual level of severity) was provided by the nuclear medicine section chief of the NYU Langone Medical Center Dr. Kent P. Friedman, based on his medical and professional experience. The assessment of presence of AD and eventual stage of severity for each subject was made after at minimum of 6 months investigation for each patient in the dataset. Dr. Friedman, utilizing the visualization obtained with the MIM (19) software from the PET/CT scan, together with the medical history, such as age, sex, previous analysis, and blood test, for the panel of the 32 patients, was capable of providing the ground truth for each subject utilized in the entire study.

**Figure 2:**
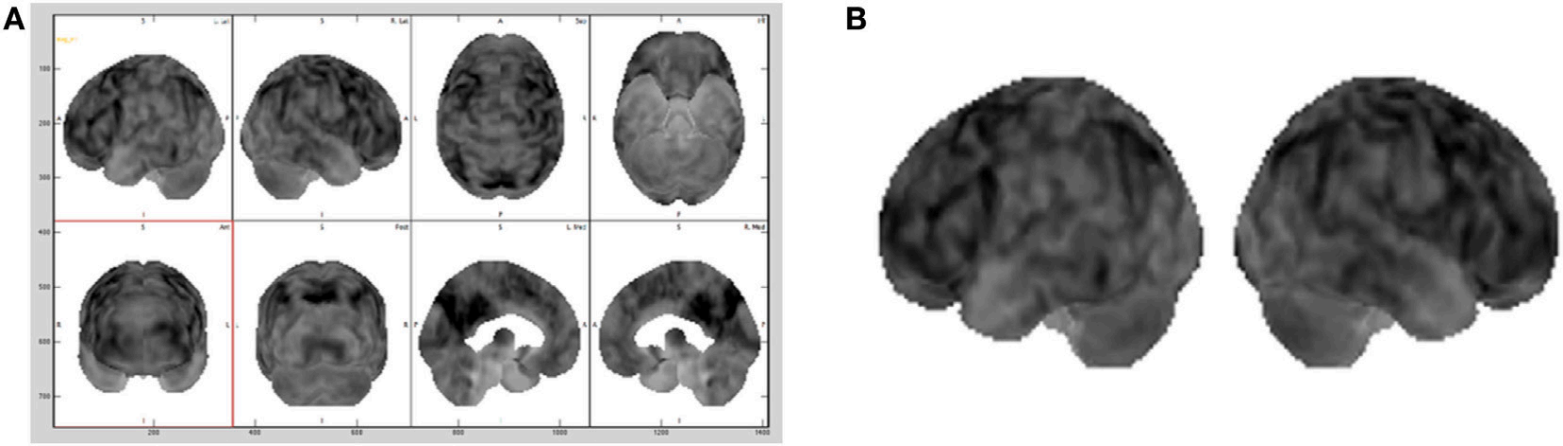
Different brains visualization using the MIM (19) software with a typical Advanced visualization **(A)** and its 3D projection **(B)** extracted.

As standard medical procedure, using the MIM (19) software, all the datasets were spatially warped to a standard brain model so that all the 32 brain scans used in the testing were spatially consistent (20). The size of each dataset was 86 × 100 × 86 voxels, with each voxel corresponding to a physical size 2 mm × 2 mm × 2 mm. These adaptations made also possible to sonify the same two-dimensional subset of data points within each three-dimensional dataset by simply choosing the same lateral slice each time for sonification. Hence, for this study the entire dataset was not utilized, but one slice per brain with its 3D projection was pulled out to be sonified. The preferable sliced chosen for the sonification was the 30th slice (from the top) that passes through representative regions of the frontal lobe, parietal lobe, and sensorimotor cortex. Consequently, for each patient, the 30th slice was selected to be the lateral slice utilized to be sonified for the purpose for this study. This corresponds to a lateral slice through the brain between 58 and 60 mm from the top edge of the dataset with size of 86 × 100 voxels. The second subset chosen for the study was a 3D projection view of the brain generated using the MIM (19) software, as illustrated in Figure 2A and in Figure 2B.

In a standard medical diagnosis physicians inspect the view of the brain generated with the MIM (19) to assess the overall picture regarding the diagnosis of a brain. Since does not allow to export the 3D projection view in the DICOM (Digital Imaging and Communication in Medicine) file format, screen captures of a gray scale representation of the 3D projection view, as shown in Figure 2B, were brought into play as base datasets for the sonification. As the 3D projection datasets are screen captures, they contain extraneous elements, such as axes, labels, and unnecessary white space. All extraneous elements were removed before being saved as a dataset, this made possible to use our sonification approach on this dataset.

**Figure 3:**
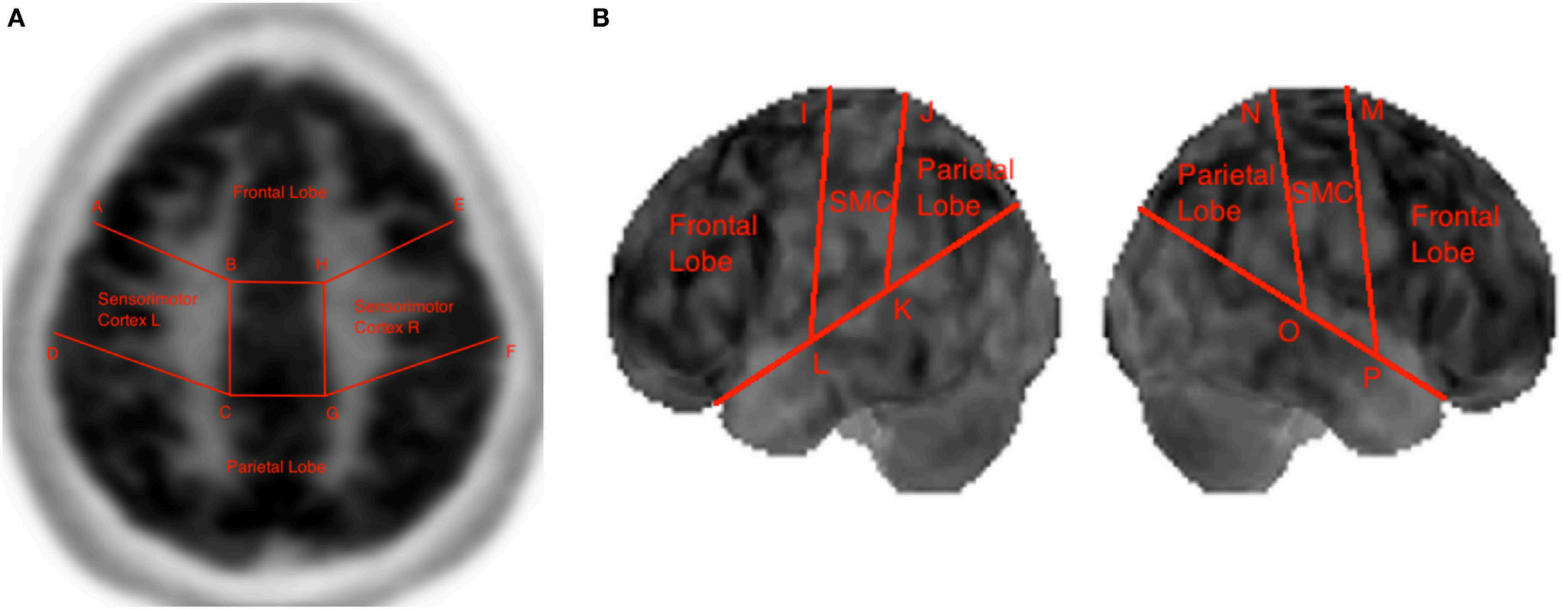
Lobe segmentation in the Triple-Tone Sonification tool of a lateral 2D slice of the brain **(A)** and of a 3D view **(B)**.

Boundary coordinates selected using the MIM (19) are shown Figure 6 and are used for lobe segmentation performed using the TTS. The visualization medical software performs its own automatic lobe segmentation of the spatially normalized brain datasets and provides segmentation information to the user in the form of the DICOM standard RTSTRUCT files. Once these coordinates are known, the datasets in output from the MIM (19), such as DICOM standard RTSTRUCT file format, goes in input into the data analysis TTS tool that performed the frontal, parietal, and SMC lobes segmentation. In the TTS tool, at this stage, the contours were approximated to straight lines and the results for lateral slices and 3D projections are shown in Figure 3A,B.

In both the lateral slice and 3D projection view remained some irrelevant data points. In PET/CT scans, data points can be irrelevant for multiple reasons, as outlined by Piper (20) and Marcus et al. (21). Some data points in the lateral slice can lie outside the actual brain area, while some other irrelevant data points consist of those that lie within the brain, but are medically irrelevant (20). The brain can for the most part be divided into white matter and gray matter (1, 22). The relevant metabolic activity for the diagnosis of AD is that of the gray matter of the brain (8). Hence, the white matter content is medically irrelevant and should not be included as part of the sonification. Therefore, all voxels whose intensity fell below a certain threshold were masked from being sonified. This threshold was set to 45% of the maximum allowable intensity, considering the bit-depth of the dataset. Hence, for datasets with a bit-depth of 15 bits, the masking threshold is set to 14,745.15 out of a maximum allowable value of 32,767.

In this data preparation, as usual procedure in diagnosis, physicians using the MIM (19), selected the two-dimensional subset of data to be sonified and the boundary coordinates for the lobes segmentation. Also as standard research medical operating routine, during this first phase of prepossessing, the multi-paradigm numerical computing environment (23) was also utilized to read the DICOM datasets in output for the MIM software through its Image Processing Toolbox and to translate and store them into arrays. All of brain scans were stored in the standard.mat format together with a text file format. The boundary coordinates for the lobes segmentation were stored in a separate text file. After the pre-processing, the following section will discuss the data analysis tool developed in this study.

### 2.2 Soniscan++

Once the medical dataset was pre-processed, it was then analyzed using a tool developed at the NYU Music Technology for the purpose of this research, named Soniscan++. This tool was introduced to translate raw medical images, stored as text files, into sound in a standardized manner that allowed reproducibility and rigor into the analysis process. The data analysis tool was developed using the object oriented programming language C++, given real-time sound synthesis speed and versatility of this programming language. The main purpose of this tool was to sonify the spatially normalized medical dataset utilizing the TTS.

**Figure 4:**
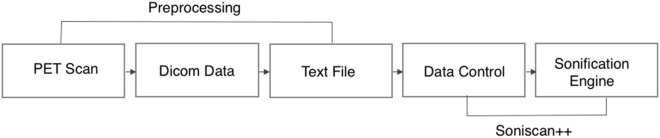
Flowchart illustration of the implementation of Soniscan++. The flowchart shows the entire process of sonification from DICOM data to the final audio.

**Figure 5:**
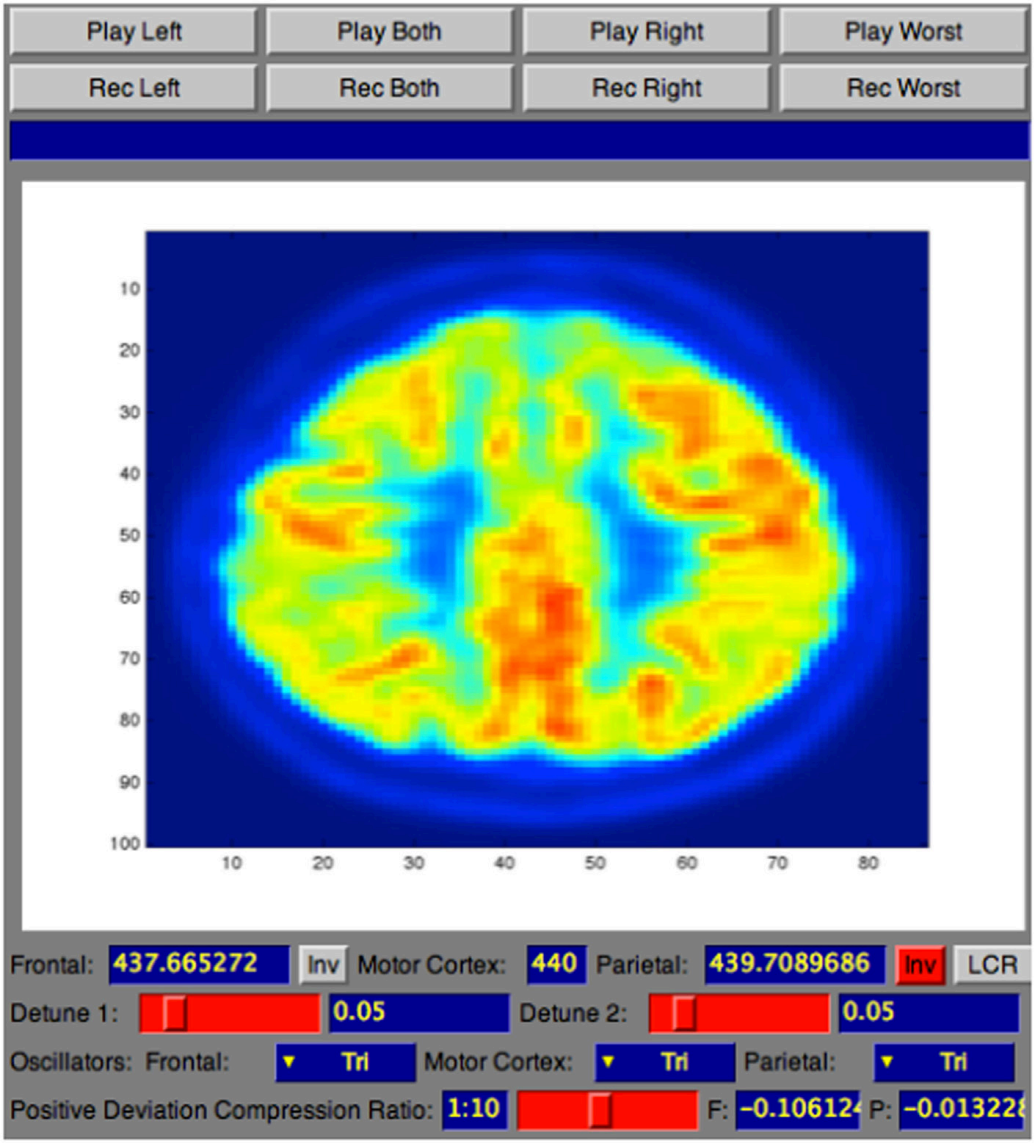
Screenshot of SuperCollider interface for sonification playback and recording.

The complete workflow of the process that translates brain scans into sound is summarized in Figure 4. As shown, SoniScan++ consists of two major blocks: the Data Control block and Sonification Engine block. Soniscan++ also output files in a.scd format so they could be easily played by a graphical user interface developed using Supercollider (24) shown in Figure 5.

**Figure 6:**
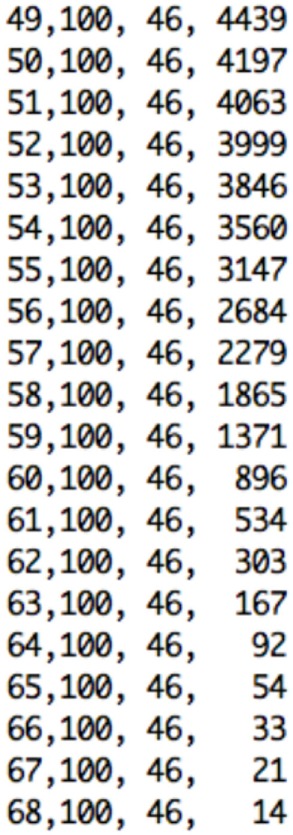
Excerpt of text format file from the pre-processing step containing x, y, and z voxels’ location, and value for the selected slice.

The Data Control block is responsible for reading the dataset in text file format as three-dimensional array, performing masking of the data, and extracting the 30th slice to be sonified. As output from the medical device the text file consists of several lines of text. As illustrated in Figure 6, each line of text refers to a single voxel, corresponding to the voxel’s x-location, y-location, z-location, and value.

Once the slice and its coordinates are extracted, the Sonification Engine block is responsible of segmenting the lobes into the three regions to be sonified, based on the given boundary coordinate points. It then removes of all irrelevant data points from the sub-datasets, using the TTS to map the difference in metabolic activity between the lobes of interest (frontal and parietal) and the reference lobe (SMC) to an easily perceivable auditory parameter. Finally, it outputs the frequencies’ differences for each slice and writes all the output into an audible format. During this stage, the Sonification Engine is also responsible for extracting randomly from the dataset the 30th slice for one participant in each category for the training session of the evaluation and avoiding that the randomly selected slices is then used for the testing phase in the evaluation process, that will be following mentioned.

**Figure 7:**
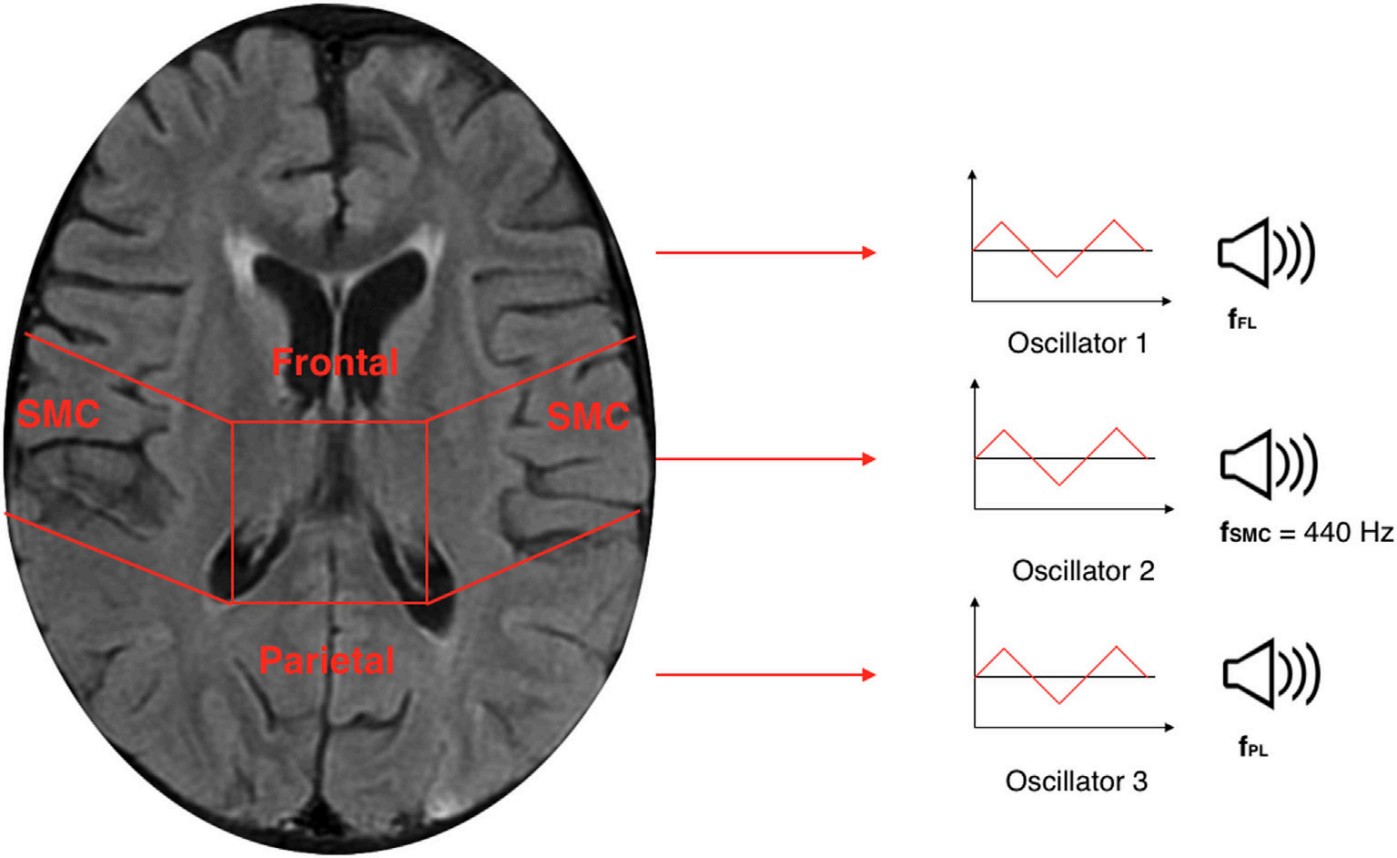
Illustration of a 30th brain slice segmented to frontal, parietal, and sensorimotor cortex to which is assigned a triangular wave oscillator to each of the three lobes.

The TTS technique, implemented in Sonification Engine block, assigns an oscillator to each of the three brain lobes of the lateral slices selected. As illustrated in Figure 7, the TTS assigns, for each slice selected, a triangular wave oscillator to the frontal lobe, parietal lobe, and sensorimotor cortex. The frequencies of these oscillators are mapped to the average metabolic activity of these regions. Specifically, to the sensorimotor cortex is given a base frequency of 440 Hz (A above middle C), and the other two lobes are deviations from that frequency. These deviation values are determined by how far the metabolic regional activity deviates from the sensorimotor cortex. Hence, differences in metabolic activity, reflecting the progress of the AD in between lobes, result in different frequencies for the oscillators. The more pronounced the difference in metabolic activity between lobes, the higher the level of AD. Thus, the more pronounced the difference between the three frequencies, the faster and more complex the beating pattern. The goal is to find different levels of beatings to indicate varying degrees of AD.

To control the range of frequencies’ deviations given the range of voxels’ deviation, a scale factor named detune factor was used for different level of AD. The frequencies corresponding to the lobes under inspection are detuned from the default frequency according to the SD of the voxels’ average intensities of frontal and parietal from the voxels’ average intensity of the reference lobe (sensorimotor cortex). To ensure that the audible parameter would not collapse to two separate beating tones when there are anomalies of equal deviation in frontal and parietal, from the default frequency, the frequency of the frontal lobe is forced to a positive deviation while the frequency of the parietal lobe is forced to a negative deviation. This difference in tones between lobes creates a beating pattern, which would be perceptible for the listener to be heard. The values for the detune factor are shown in Table 1.

**Table 1:** List of detune factor values for normal brain, mild AD, moderate AD, and severe AD.

Brain type	Detune factor
Normal brain	0.05
Mild AD	0.10
Moderate AD	0.15
Severe AD	0.20

Therefore, given the detune factor, the frequency of the frontal (*f_FL_*) and parietal (*f_PL_*) lobes were determined as follows:

$$f_{FL}=f_{default}*\left(1+DF*\left|\frac{av_{FL}-av_{SMC}}{av_{SMC}}\right|\right)=f_{default}*\left(1+DF*|\Delta_{FL}|\right)$$

$$f_{PL}=f_{default}*\left(1+DF*\left|\frac{av_{PL}-av_{SMC}}{av_{SMC}}\right|\right)=f_{default}*\left(1+DF*|\Delta_{PL}|\right).$$

In the equations above, the relative deviation of average intensity (*av_FL_* and *av_PL_*) with respect to the average intensity of the sensorimotor cortex (*av_S_MC*) are linearly mapped to the relative deviation of the oscillator frequency with respect to the oscillator’s base frequency (*f_default_*) through the detune factor (DF) coefficient.

In conclusion, these differences in oscillation between the three oscillators result in beating patterns that will be heard from the two controlled group during the evaluation process. [You can listen to the four sonified sound samples for a subject not affected by AD (Audio S1 in Supplementary Material), for a patient mildly affected by AD (Audio S2 in Supplementary Material), for a subject moderately affected by AD (Audio S3 in Supplementary Material), and for a subject severely affected by AD (Audio S4 in Supplementary Material).]

## 3 Evaluation

The implementation features of TTS developed in the Soniscan++ tool were analyzed in the previous section. The TSS produced differences in oscillators resulting in hearable beating patterns that can tackle differences across the four levels of AD cases presented in this study. The technique was validated among two groups of listeners against the ground truth provided by Dr. Friedman and evaluated on how effectively it allowed those listeners to distinguish between brains of different levels of AD with higher accuracy that using the visualizations only.

The first round of testing was presented to trained musical ears of audio professionals, while the second round of testing to physicians in the field of radiology. The purpose of the first round was to validate the effectiveness of the TTS in accurate categorizations of the beating pattern. The main goal of the second round testing was to determine the accuracy and intra-reader consistency in the diagnosis process. Both testing groups forerun by a training session. The purpose of this warming up session was to let listeners familiarize with the sonification tool before the actual analysis.

During the training session were randomly selected four subjects for each of the four categories of AD from the dataset. These four slices, one for each of the subject selected, were extracted from the Sonification Engine implemented in the Soniscan++ and not used in the testing process. For each subject, it was used the orthogonal 2-dimensional view (basic visual) of the 30th slice and the related 3-dimensional view (advanced visual), and basic and advanced visualizations were played always one after the other for the selected slice. The selected training samples, for basic and advanced visuals, were played for each of the four categories including normal, mild, moderate, and severe. These sonifications were played for 30 s each, with approximately 1 min break one after the other, while participants were looking at the lateral and advanced visualizations related to that particular sonification. The order at which they were presented was always structured with the basic visualization first and the advanced visualization following the basic for each of the four AD level, presented from normal to severe. This training session was performed to introduce them the sound related to each of the four AD categories.

Following the training session, a testing session was presented to the two groups of listeners. Also during this session, 2-dimensional (basic) and 3-dimensional (advanced) visualizations were utilized one after the other for the selected slices in each of the four AD categories. Only this time, the order at which these slices were presented, with respect to the AD level of disorder, was randomized in order to test the sonification tool added to the basic and advanced visualizations. All the sonifications were forerun by a standard visualization without sonification added. This was done to mimic a diagnosis scenario. Overall, the order at which was presented the dataset to the two groups of listeners was divided into four steps. During the first round, it was presented the two visualizations, basic followed by advanced, without any sonification. During the second round for each of the two visualizations was added a sonification related to the particular visualization selected. In the following subsection, the two rounds are presented one after the other, as it was during the evaluation procedure.

### 3.1 Testing: Round One

During this round, testing occurred over four sessions, including basic and advanced visualizations with and without sonification. Here, the evaluation was performed into two sections, namely “coarse categorization” and “fine categorization.” For the “coarse categorization,” four classes, numbered from one to four, were chosen to correspond to the diagnosis of four different levels of AD (normal, mild, moderate, severe). In the “fine categorization,” participants were instead asked to classify each sonification into a seven step categorization, numbered 1, 1.5, 2, 2.5, 3, 3.5, and 4. In this second case, subjects were instructed to rate both cases that aligned with the training cases to the integer-valued categories (1, 2, 3, and 4), corresponding to AD levels, and cases that may be interpreted as lying between training cases as the fractional-value categories (1.5, 2.5, and 3.5). Each subject during each section had to categorize the presented sonification into one of the four coarse numbers as well as into one of seven fine categories. This was done to investigate whether the TTS provided a finer gradation that improved diagnosis accuracy in distinguishing more accurate or consistent results.

First, a basic visualization of the 30th orthogonal slice of the brain was presented, randomly selected from the dataset. Then, for the same subject, the Sonification Engine implemented in the Soniscan++ selected the relative 3-dimensional visualization. These two sessions were initially shown for the entire dataset without any sonification added and it was asked to the testers to indicate a category one of the four coarse numbers as well as into one of seven fine categories for the entire dataset. After 10 min, they completed the first session, the second section, in which the sonification was added for basic and advanced visual was added. The Sonification Engine randomly presented the same subjects and the same visualizations, only this time 30-s sonification played along with basic and advanced visuals for all the subjects in the dataset.

This subjective listening test was presented to five testers, all graduate students, and faculty members of the Music Technology program at the NYU Steinhardt. This group was chosen to first evaluate the performances of the TTS technique because of their musically trained ears, providing a technical validation before proceeding with the evaluation of radiologists during the second round of testing. Results are reported in the following section.

### 3.2 Testing: Round Two

As for the first round, also the second round testing took place over two sessions. During these sessions, the Sonification Engine randomly selected a two-dimensional lateral slice and the related three-dimensional projection of the same brain, for each of the 32 de-identified brains in the dataset. In the first session, basic and advanced visualizations were presented without sonification; while in the second session, the sonification was added for both visualizations and played along for each subject in the dataset. Here, the evaluation was divided into four clusters that corresponded to the four different levels of brain in the dataset, including normal, mild, moderate, and severe. All the sessions were set up to mimic the diagnosis process that the radiologist would normally undertake. Therefore, each radiologist during each of the two sessions performed the diagnosis looking at the left hemispheres and right hemispheres of the frontal and the parietal lobes assessing a level of disease with respect to brain presented.

This second round of testing was performed by two radiologists from the NYU Langone Medical Center, one highly experienced radiologist and one less experienced radiologist. The physicians were given no information about the test case, the patient’s gender, the age, or the medical history. The first session included the standard visualization of the entire dataset without any sonification. After 10 min from the first session, the second stage tested the TTS performance in enhancing distinguishability among different level of AD. Each session included diagnoses of the same 32 de-identified PET scan cases. The order of presenting the sonification was randomized across diagnoses. Results are shown and discussed in the following session.

## 4 Results

### 4.1 Results: Round One

This first group of participants consisting of trained ears of musical professionals aimed to classify the results in “coarse categorization” and “fine categorization” for each of the four categories of AD level, including normal, mild, moderate, and severe. The accuracy of response in the “coarse categorization” responses were matched against the ground truth, while for the “fine categorization” responses were either matched against the ground truth or lied at a distance of 0.5 from it.

Results from Table 2 present the percentage of accuracies for each participant in discriminating between cases using the “coarse categorization,” and results in Table 3 illustrate the same accuracy in distinguishing between different AD levels using the “fine categorization.” In the case of coarse categorization, the responses to a pair of duplicate test cases are said to be consistent if both cases were given the same response by the participant. In the case of fine consistency, the responses to a pair of test cases are said to be consistent if both cases were given responses that differ by no more than 0.5. A side-by-side comparison of participant accuracies is given in Table 4 for coarse and fine sections. In the case of coarse categorization, mimicking the diagnosis procedure of AD, participant displayed an overall accuracy of 87%.

**Table 2:** Accuracy of participants in differentiating using the “coarse categorization” across four categories of Alzheimer’s dementia.

Participants	Cat.1 (%)	Cat.2 (%)	Cat.3 (%)	Cat.4 (%)
P1	100	88	81	88
P2	88	94	38	94
P3	94	100	81	100
P4	100	94	75	100
P5	94	94	44	100

**Table 3:** Accuracy of participants in differentiating using the “fine categorization” across four categories of Alzheimer’s dementia.

Participants	Cat.1 (%)	Cat.2 (%)	Cat.3 (%)	Cat.4 (%)
P1	88	88	81	88
P2	100	100	56	100
P3	100	100	100	100
P4	94	100	88	100
P5	100	100	69	100

**Table 4:** Comparison of participants’ accuracy in discriminating different level of dementia using coarse and fine categorizations.

Participant	Coarse Categ. (%)	Fine Categ. (%)
P1	89	86
P2	78	89
P3	94	100
P4	92	95
P5	83	92

### 4.2 Results: Round Two

The previous round validated the technique providing its capability to yield accurate categorizations of the beating patterns. However, that testing round was done on the trained musical ears of audio professionals. The goal of the second round was to determine the validity of the technique on radiologists.

At the end of the final testing session, physicians were given a survey with questions. The purpose of the survey was to investigate if they found the sonification helpful in the diagnoses process. The questionnaire is shown in Table 5 and their answers were given on a scale from 1 to 5, where 1 = strongly disagree, 2 = disagree, 3 = somewhere in the middle, 4 = agree, and 5 = strongly agree. As shown in Table 5, the radiologists found the sonification technique although not pleasant, helpful in the diagnosis process especially when it came to discerning between different levels of AD in solidifying the validity of the overall process.

**Table 5:** Physician questionnaire after the training and testing to evaluate the performances of the “Triple-tone” sonification technique.

Questions	Phys. high exp.	Phys. low exp.
The sonification was tiring to listen to and induced fatigue	2	1
The sonification was pleasant	2	2
The sonification provided additional information that the visual display did not	3	5
The sonification was helpful in discerning between	–	–
(a) normal and mild cases	5	4
(c) mild and moderate cases	5	4
(e) mild and severe cases	4	4
The sonification made me more certain about my diagnosis	4	4

Then radiologists underwent to four distinct sessions including basic and advanced visualization with and without the sonification for the 32 de-identified brains. Since AD can be asymmetrical and the aim of this session was to mimic a standard diagnosis procedure, it was necessary to dissect these regions by hemispheres, analyzing the lefts and the rights. So for each one of the 32 unidentified brain scans of patients, physicians analyzed four parts of the brain: left frontal (LF), left parietal (LP), right frontal (RF), and right parietal (RP). The results were then compared to the ground truth provided by Dr. Friedman.

**Figure 8:**
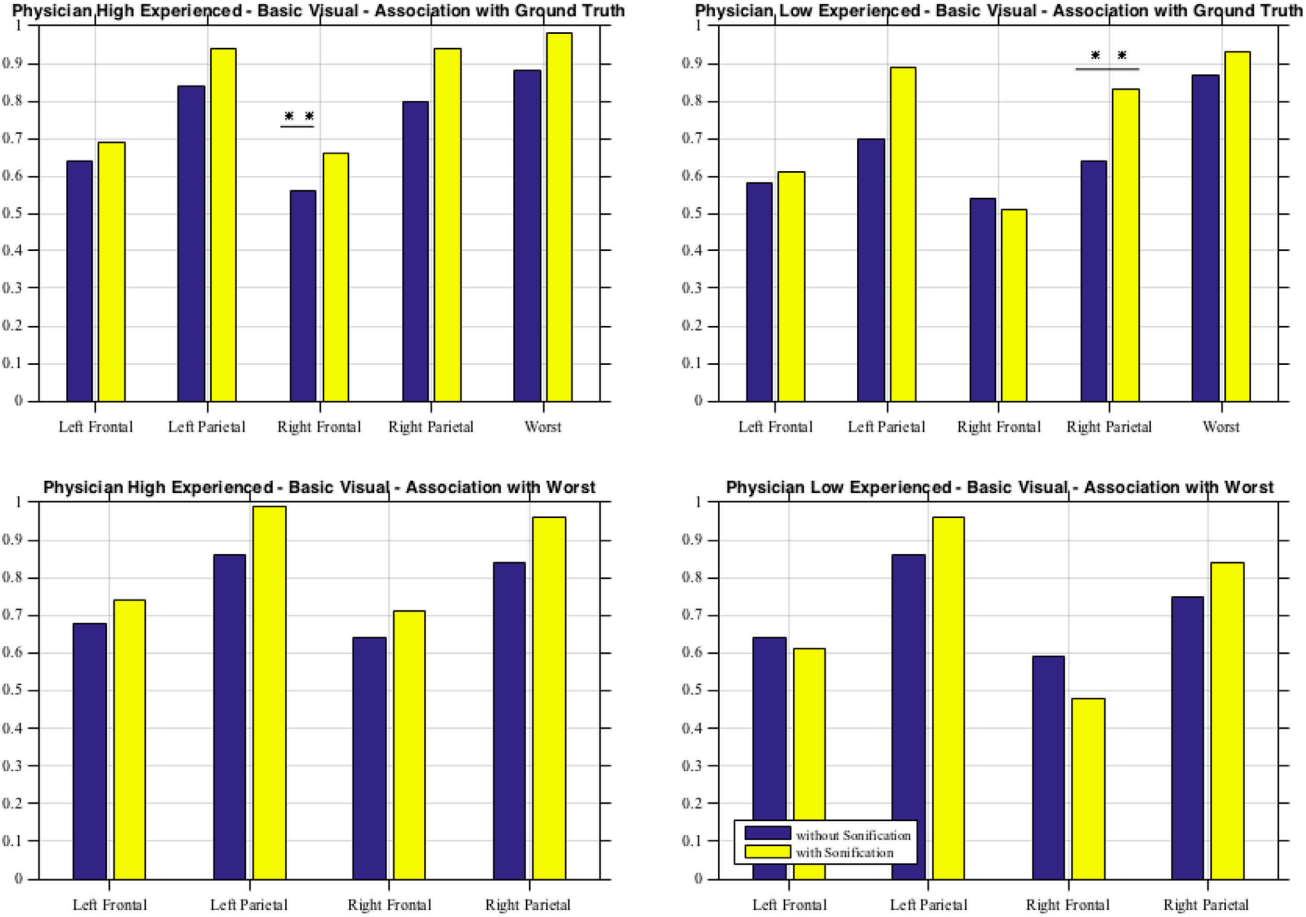
Basic visual with and without sonification for the two physicians in association with the ground truth and with the worst scores.

As shown in Table 6 for each combination of two physicians and four sessions, there was a significant positive correlation between the ground and the frontal, parietal and worst regions. Comparing basic and advanced inspections with and without sonification, a significant increase of the correlation is shown in Table 6 and also illustrated in Figure 8.

**Table 6:** Pearson’s correlation for association with the ground truth, ***significant at p < 0.001, ** significant at p < 0.005, * significant at p < 0.5, for Physician 1 (high experienced).

Sess.	LF	LP	RF	RP	W
**Physician 1 (high experienced)**					
0 Basic Visual	0.64***	0.84***	0.56**	0.8***	0.88***
1 Adv. Visual	0.39*	0.76***	0.37*	0.74***	0.81***
2 Basic Visual + Sonif.	0.69***	0.94***	0.66***	0.94***	0.98***
3 Adv. Visual + Sonif.	0.68***	0.79***	0.67***	0.81***	0.86***
**Physician 2 (low experienced)**					
0 Basic Visual	0.58***	0.70***	0.54**	0.64***	0.87***
1 Adv. Visual	−0.15	0.00	−0.01	0.12	0.03
2 Basic Visual + Sonif.	0.61***	0.89***	0.51**	0.83***	0.93***
3 Adv. Visual + Sonif.	0.58**	0.85***	0.48*	0.79**	0.95***

Since the highest significant correlation was always between the ground truth and the worst scores, accuracy was assessed in terms of concordance between the truth and worst results and the correlation for the association of the regional scores with worst cores is presented in Table 7. In Table 7, all correlations were significant (p < 0.01). The worst scores were most highly correlated with the scores from left parietal region, highlighted in bold. The result from these tables for basic visuals is shown in Figure 8.

**Table 7:** Pearson’s correlation for association with worst scores.

Sess.	LF	LP	RF	RP
**Physician 1 (high experienced)**				
Basic Visual	0.68	**0.86**	0.64	0.84
Advanced Visual	0.57	**0.97**	0.55	0.91
Basic Visual + Sonification	0.74	**0.99**	0.71	0.96
Advanced Visual + Sonification	0.87	**0.96**	0.85	0.92
**Physician 2 (low experienced)**				
Basic Visual	0.64	**0.86**	0.59	0.75
Advanced Visual	0.57	**0.97**	0.55	0.91
Basic Visual + Sonification	0.61	**0.96**	0.48	0.84
Advanced Visual + Sonification	0.52	**0.85**	0.44	0.77

## 5 Discussion and Conclusion

Effort has been made in the recent years to explore alternative representation methods to improve the accuracy and consistency in the diagnosis process. Several studies have explored the possibility of aided diagnosis tools using auditory feedbacks. In this work, we present a novel Triple-Tone Sonification technique to analyze PET scans of brains with different levels of Alzheimer’s dementia. The model, presented and evaluated using subjective listening test, provides a supportive tool in the diagnosis of Alzheimer’s when the metabolic activity is under inspection. Participants involved in this study were professional musician with trained musical ears and physicians in the field of radiology.

Results of the evaluation of the sonification method among the first subjective listening group indicate that participants with musically trained ears were able to categorize the sonifications with an average accuracy of 87% using “coarse categorization.” The overall accuracy in diagnostic categorization improved from 87.19 to 92.5% when a finer gradation of categorization was utilized. This indicates that there exist sonifications generated by this technique that place a brain scan “in between” two categories when evaluated by a listener. In the case of “coarse categorization,” the in-between scans were perceptually quantized into one of the coarse categories by the participant. In the case of “fine categorization,” the participant was able to successfully categorize the sonification as an in-between case.

From the second subjective listening testing, as illustrated in Tables 6 and 7 and Figure 8, results have shown that there is a significant positive correlation between the ground truth and the frontal, parietal, and worst regions. During the basic visual testing, one physician achieved an accuracy of 56–88% while the other performed at an average of 50–55%. Comparing basic and advanced inspections with and without sonification, a significant increase is shown in Figure 8. For each session, from an average improvement of about 10%, for the more severe cases, physicians results improved in accuracy up to about 30% with the sonification added. These results show that, using the TTS method, medical researchers can utilize an additional way of interpreting data that can help detecting something that may be missed with traditional visual inspection.

Additional ways of interpreting and representing data have been largely explored, with a particular attention in the auditory feedback in works, such as Hermann (25), Hermann and Hunt (26), and Hunt and Hermann (27). Sonification provides new tools for recognizing patterns and analyzing data, extending the process of discovery in a number of diverse fields. In this work, a new sonification technique to meliorate the diagnosis of Alzheimer’s dementia has been presented. The TTS technique implemented in this study has been evaluated on trained and untrained ears. The promising results acquired in this analysis and discussed in the previous section highlight more possibilities in helping identifying different stages of AD in the diagnosis process. Under the first evaluation of accuracy and consistency, the TTS technique validates its capabilities in allowing a finer gradation of providing accurate diagnosis. In this scenario, intra-reader consistency of categorization with sonification is superior to that of visualization tools alone. During the second round inspection, it was extended the influence of the sonification in the diagnosis of brain scans among two physicians. Here, intra- and inter-reader variability in accurate distinguishability of different levels of AD increased with the sonification added for both experienced and inexperienced physicians. To evaluate this technique, the correlation between each evaluation method has been proposed in this study. The correct diagnosis based on the ground truth has been computed to measure if sonification helped improve accuracy in the diagnosis.

A possible future scenario for the technique would be working with 3D audio and enhancing the analysis and/or experience of diagnosing AD. This sonification technique can be also used to augment existing visualization imaging techniques, such as MRI, in order to bring considerable improvements to medical diagnosis area. Furthermore the detune factor is currently manually changed according to the ground truth of the case. This is acceptable as a validation of the sonification technique, but not for an objective data-driven sonification model. For future investigations, this detune factor will have to be mapped to an appropriate feature of the data. This would ensure that the sonification is purely data-driven and remains completely objective, while still allowing for a variable detune factor to increase the severity of sonification with severity of disease.

The validation of the efficacy of the TTS tool proposed in this article provides evidence through higher accuracy in diagnosis among the two subjective listening groups when this sonification is used. The technique, successfully validated, with future investigations, can become an integral part of a physician’s diagnostic toolkit and may open up in helping with AD diagnosis.

## Ethics Statement

This study was carried out in accordance with the recommendations of the Division of Medical Ethics at NYU with written informed consent from all subjects. All subjects gave written informed consent in accordance with the Declaration of Helsinki. The protocol was approved by the Division of Medical Ethics at NYU.

## Author Contributions

LG was a visiting student at the NYU Langone Medical Center and researcher involved in the study at the Music Technology Department of NYU Steinhardt. AR was the principal investigator of the project at the Music Technology Department of NYU Steinhardt.

## Conflict of Interest Statement

The authors declare that the research was conducted in the absence of any commercial or financial relationships that could be construed as a potential conflict of interest.

## Supplementary Material

The p-value is the probability that it is found the current result if the correlation coefficient is zero (null hypothesis). If this probability is lower than the conventional 5% (p < 0.05), the correlation coefficient is called statistically significant.

Alzheimer’s dementia can affect patients’ brains in dissimilar and asymmetrical forms. In some cases, the decrease in metabolic activity manifests itself uniformly across lobes, while in others the same decrease is more concentrated in certain regions of the lobes (28). To take into account this asymmetry, the datasets after being segmented into lobes are segmented into left and right halves. Diagnosis of the stage of Alzheimer’s is performed according to the worst affected area of the brain (29). In this manner, in case of asymmetries, left and right halves of the brain can be rendered to two separate sonifications, thereby allowing the physician to identify the “worse half” and make a diagnosis that accurately represents the stage of AD. A quantitative estimation is also performed to automatically identify the worse half, as follows:

if(|ΔFL|+|ΔPL|) of left>(|ΔFL|+|ΔPL|) of right=>worst half=left halfelse worst half=right half.

## References

[1] Berchtold N Cotman C Evolution in the conceptualization of dementia and Alzheimer’s disease: Greco-roman period to the 1960s. Neurobiol Aging (1998) 19(3):173–89. 9661992 10.1016/s0197-4580(98)00052-9

[2] McKhann G Drachman D Folstein M Katzman R Price D Stadlan EM Clinical diagnosis of Alzheimer’s disease: report of the nincds-adrda work group under the auspices of department of health and human services task force on Alzheimer’s disease. Neurology (1984) 34(7):939–44. 10.1212/WNL.34.7.939 6610841

[3] Meltzer C Zubieta J Brandt J Tune L Mayberg H Frost J Regional hypometabolism in Alzheimer’s disease as measured by positron emission tomography after correction for effects of partial volume averaging. Neurology (1996) 47(2):454–61. 10.1212/WNL.47.2.454 8757020

[4] Frackowiak R Pozzilli C Legg N Boulay GD Marshall J Lenzi G Regional cerebral oxygen supply and utilization in dementia. A clinical and physiological study with oxygen-15 and positron tomography. Brain (1981) 104(4):753–78. 10.1093/brain/104.4.753 6976816

[5] Miller-Thomas MM Sipe AL Benzinger TLS McConathy J Connolly S Schwetye KE. Multimodality review of amyloid-related diseases of the central nervous system. Radiographics (2016) 36(4):1147–63. 10.1148/rg.2016150172 27399239 PMC4976469

[6] Rice SL Kent FP. Clinical pet-mr imaging in breast cancer and lung cancer. PET Clin (2016) 11(4):387–402. 10.1016/j.cpet.2016.05.008 27593245 PMC5538357

[7] Kok TY Padhy AK The conundrum of pet/mr. World J Nucl Med (2012) 11(1):1–2. 10.4103/1450-1147.98717 22942773 PMC3425221

[8] Illán IA Górriz JM Ramírez J Salas-Gonzalez D López MM Segovia F 18f-fdg pet imaging analysis for computer aided Alzheimer’s diagnosis. Inf Sci (2011) 181(4):903–16. 10.1016/j.ins.2010.10.027

[9] Stockbridge H Lewis D Eisenberg B Lee M Schacher S van Belle G Brain spect: a controlled, blinded assessment of intra-reader and inter-reader agreement. Nucl Med Commun (2002) 23(6):537–44. 10.1097/00006231-200206000-00005 12029208

[10] Rossiter D Ng W-Y A system for the complementary visualization of 3d volume images using 2d and 3d binaurally processed sonification representations. Proceedings of the 7th Conference on Visualization ’96 Los Alamitos, CA: IEEE Computer Society Press (1996). p. 351–4.

[11] Hermann T Hunt A Neuhoff JG The Sonification Handbook. Berlin: Logos Publishing House (2011).

[12] Walker BN Kramer G Sonification design and metaphors: comments on Walker and Kramer. ACM Trans Appl Percept (2005) 2(4):413–7. 10.1145/1101530.1101535

[13] Walker BN Mauney LM Universal design of auditory graphs: a comparison of sonification mappings for visually impaired and sighted listeners. ACM Trans Access Comput (2010) 2(3):16 10.1145/1714458.1714459

[14] Polli A “Atmospherics/weather works”: a spatialized meteorological data sonification project. Leonardo Music J (2005) 38(1):31–6. 10.1162/leon.2005.38.1.31

[15] Polli A Heat and the heartbeat of the city: sonifying data describing climate change. Leonardo Music J (2006) 16(1):44–5. 10.1162/lmj.2006.16.44

[16] Baier G Hermann T The sonification of rhythms in human electroencephalogram. In: Brazil E, editor. Proceedings of the 10th International Conference on Auditory Display Sydney: Springer-Verlag (2004). p. 6–9.

[17] Kagawa T Kudo H Tanoue S Kiyosue H Mori H Nishino H A supporting method of medical imaging diagnosis with sonification. Proceedings of the 2002 International Conference on Auditory Display Kyoto: IEEE (2012). p. 699–704.

[18] Ballora M Pennycook B Ivanov PC Glass L Goldberger AL Heart rate sonification: a new approach to medical diagnosis. Leonardo Music J (2004) 37(1):41–6. 10.1162/002409404772828094

[19] MIM Software Inc. (2014). Available from: https://www.mimsoftware.com/

[20] Piper J Quantitative comparison of spatial normalization algorithms for 3d pet brain scans. J Nucl Med (2007) 48(2):403–8. 10.1002/hbm.10047

[21] Marcus C Whitworth P Surasi D Subramaniam R Pet/ct in the managements of thyroid cancers. Am J Roentgenol (2014) 202(6):1316–29. 10.2214/AJR.13.11673 24848831

[22] Nelson A Piper J Friedland R Freeman B Probabilistic human brain atlas for functional imaging: comparison to single brain atlases. J Nucl Med (2007) 48(2):3508–26.

[23] MATLAB. Version 7.10.0 (R2010a) (2010). The MathWorks Inc.

[24] Wilson S Cottle D Collins N The SuperCollider Book. Cambridge: The MIT Press (2011).

[25] Hermann T Taxonomy and definitions for sonification and auditory display. In: Susini P Warusfel O, editors. Proceedings of the 14th International Conference on Auditory Display Paris, France: IRCAM (2008). p. 24–7.

[26] Hermann T Hunt A Guest editors’ introduction: an introduction to interactive sonification. IEEE MultiMedia (2005) 12(2):20–4. 10.1109/MMUL.2005.26

[27] Hunt A Hermann T The importance of interaction in sonification. In: Barrass S Vickers P, editors. Proceedings of the 10th International Conference on Auditory Display Sydney: ICAD (2004). p. 1–8.

[28] Geroldi C Akkawi NM Galluzzi S Ubezio M Binetti G Zanetti O Temporal lobe asymmetry in patients with Alzheimer’s disease with delusions. J Neurol Neurosurg Psychiatry (2000) 69(2):187–91. 10.1136/jnnp.69.2.187 10896691 PMC1737042

[29] Rosen A An introduction to cognitive aging and dementia: a clinical neuropsychologist’s perspective. Proceedings of the 2016 CHI Conference Extended Abstracts on Human Factors in Computing Systems San Jose: ACM (2016). p. 948–51.

